# Biotechnical development of genetic addiction risk score (GARS) and selective evidence for inclusion of polymorphic allelic risk in substance use disorder (SUD)

**DOI:** 10.15761/JSIN.1000221

**Published:** 2019-12-19

**Authors:** K Blum, A Bowirrat, D Baron, L Lott, JV Ponce, R Brewer, D Siwicki, B Boyett, MC Gondre-Lewis, DE Smith, Thanos Panayotis K, S Badgaiyan, M Hauser, L Fried, Roy A., BW Downs, RD Badgaiyan

**Affiliations:** 1 Western University Health Sciences Graduate School of Biomedical Sciences, Pomona, CA, USA; 2 Department of Precision Behavioral Management, Geneus Health, San Antonio, TX, USA; 3 Division Addiction Services, Dominion Diagnostics, LLC, North Kingston, RI, USA; 4 Division of Nutrigenomics, Victory Nutrition International. Inc. Lederach, PA, USA; 5 Divion of Neuroscience & Addiction Research, Pathway HealthCare, LLC, Birmingham, AL; 6 Institute of Psychology, ELTE Eötvös Loránd University, Budapest, Hungary; 7 Department of Psychiatry, University of Vermont, Burlington, VM. USA; 8 Centre for Genomics and Applied Gene Technology, Institute of Integrative Omics and Applied Biotechnology, Nonakuri, Purba Medinipur, West Bengal, India; 9 Department of Psychiatry, Wright State University Boonshoft School of Medicine and Dayton VA Medical Centre, Dayton, OH, USA; 10 Departments of Clinical Neuroscience and Population Genetics, Interdisciplinary Center (IDC) Herzliya, Department of Neuroscience, Israel; 11 National Human Genome Center, Howard University, Washington DC, USA; 12 Departments of Anatomy, and Psychiatry & Behavioral Sciences, Howard University College of Medicine, Washington DC, USA; 13 Department of Pharmacology, University of California San Francisco School of Medicine, San Francisco, USA; 14 Behavioral Neuropharmacology and Neuroimaging Laboratory on Addictions, Research Institute on Addictions, University at Buffalo, Buffalo, NY, USA; 15 Transformations Treatment Center, Del-Ray Beach, FL, USA; 16 Department of Psychiatry, Tulane University School of Medicine, New Orleans, LA, USA; 17 Department of Psychiatry, Ichan School of Medicine at Mount Sinai, New York, NY., USA; 18 Department of Psychiatry, South Texas Veteran Health Care System, Audie L. Murphy Memorial VA Hospital, San Antonio, TX, USA; 19 Long School of Medicine, University of Texas Medical Center, San Antonio, USA^2^Instituto Nacional de Neurología y Neurocirugía

**Keywords:** substance use disorder (sud), genetic addiction risk score (gars®), dopamine. brain reward circuitry, precision addiction management (pam®), reward deficiency syndrome (rds)

## Abstract

Research into the neurogenetic basis of addiction identified and characterized by Reward Deficiency Syndrome (RDS) includes all drug and non-drug addictive, obsessive and compulsive behaviors. We are proposing herein that a new model for the prevention and treatment of Substance Use Disorder (SUD) a subset of RDS behaviors, based on objective biologic evidence, should be given serious consideration in the face of a drug epidemic. The development of the Genetic Addiction Risk Score (GARS) followed seminal research in 1990, whereby, Blum’s group identified the first genetic association with severe alcoholism published in JAMA. While it is true that no one to date has provided adequate RDS free controls there have been many studies using case –controls whereby SUD has been eliminated. We argue that this deficiency needs to be addressed in the field and if adopted appropriately many spurious results would be eliminated reducing confusion regarding the role of genetics in addiction. However, an estimation, based on these previous literature results provided herein, while not representative of all association studies known to date, this sampling of case- control studies displays significant associations between alcohol and drug risk. In fact, we present a total of **110,241** cases and **122,525** controls derived from the current literature. We strongly suggest that while we may take argument concerning many of these so-called controls (e.g. blood donors) it is quite remarkable that there are a plethora of case –control studies indicating selective association of these risk alleles ( measured in GARS) for the most part indicating a hypodopaminergia. The paper presents the detailed methodology of the GARS. Data collection procedures, instrumentation, and the analytical approach used to obtain GARS and subsequent research objectives are described. Can we combat SUD through early genetic risk screening in the addiction field enabling early intervention by the induction of dopamine homeostasis? It is envisaged that GARS type of screening will provide a novel opportunity to help identify causal pathways and associated mechanisms of genetic factors, psychological characteristics, and addictions awaiting additional scientific evidence including a future meta- analysis of all available data –a work in progress.

## Introduction

### History of Reward Deficiency Syndrome (RDS) development

Research into the neurogenetic basis of addiction identified and characterized by Reward Deficiency Syndrome (RDS) [1] includes all drug and non-drug addictive, obsessive and compulsive behaviors. We are proposing herein that a new model for the prevention and treatment of RDS behaviors based on objective biologic evidence should be given serious consideration in the face of a drug epidemic [2]. Currently, research directed toward improving treatment for highly drug-dependent patients in underserved populations represents one example of adoption of this bold concept and is under study through a NIH grant [3]. The grant explores utilization of the Genetic Addiction Risk Score (GARS) and the neuronutrient pro-dopamine regulator KB220.

The development of GARS followed seminal research in 1990, whereby, Blum’s group identified the first genetic association with severe alcoholism published in JAMA [4]. The non-invasive GARS test identifies and measures the total number of risk alleles of genes and catabolic enzymes affecting an individual’s neurochemical hypodopaminergic function and has been associated in hundreds of studies with SUD [5].

According to the American Society of Addiction Medicine (ASAM), addiction is a “primary, chronic disease of brain reward, motivation, memory and related circuitry.” This defintion communicates the many effects of addiction, but the factors that can increase the risk of addiction are also varied, including: genetic predisposition, comorbid psychiatric conditions, and certain at-risk environments. Addiction is a broad term that can refer to substance addiction (e.g., opioids, prescription drugs) and non-substance addiction (e.g., thrill seeking, gambling) [6].

**Figure 1.**
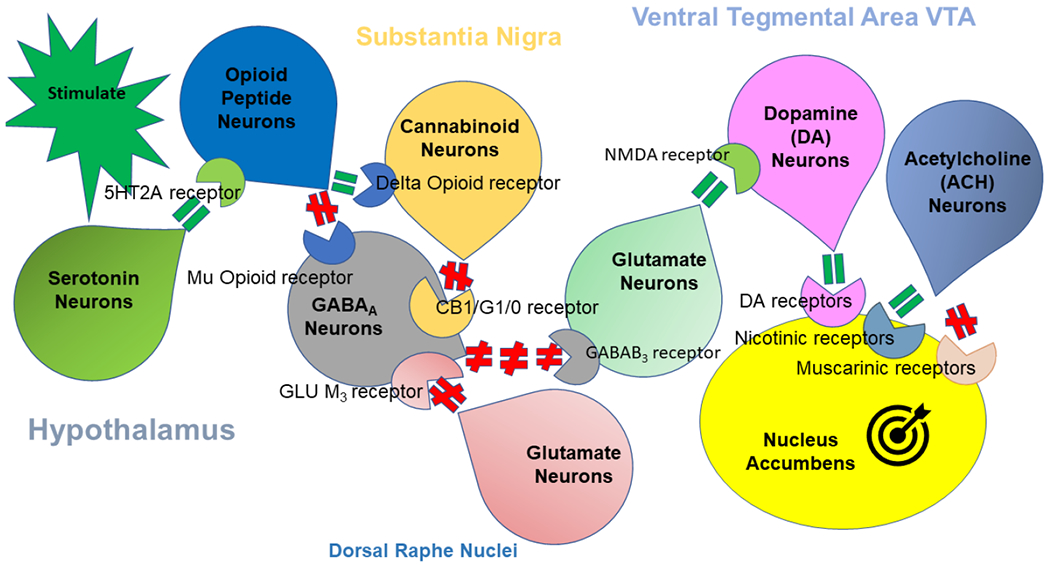
The brain reward cascade (BRC). Figure1 illustrates the interaction of at least six principal neurotransmitter pathways involved in the Brain Reward Cascade (BRC). In the hypothalamus, environmental stimulation causes the release of serotonin, which in turn via, for example, 5HT-2a receptors activate (green, equal sign) the subsequent release of opioid peptides also in the hypothalamus. Then, in turn, the opioid peptides having two distinct effects possibly via two different opioid receptors: A) inhibits (red hash sign) through the mu-opioid receptor (possibly via enkephalin) and projects to the Substania Nigra to GABA_A_ neurons B) stimulates (green equal sign) Cannabinoid neurons (e.g., Anandamide and 2-archydonoglcerol) through Beta –Endorphin linked delta receptors, which in turn inhibits GABA_A_ neurons at the substania nigra. Cannabinoids primarily 2-archydonoglcerol, when activated, can also indirectly disinhibit (red hash sign) GABA_A_ neurons in the Substania Nigra through activation of G1/0 coupled to CB1 receptors. Similarly, Glutamate neurons located in the Dorsal Raphe Nuclei (DRN) can indirectly disinhibit GABA_A_ neurons in the Substania Nigra through activation of GLU M_3_ receptors (red hash sign). GABA_A_ neurons, when stimulated, will, in turn, powerfully (red hash signs) inhibit VTA glutaminergic drive via GABAB _3_ neurons. Finally, Glutamate neurons in the VTA will project to dopamine neurons through NMDA receptors (green, equal sign) to preferentially release dopamine at the Nucleus Accumbens (ACH)shown as a bullseye indicating euphoria (a *wanting* response)

The mesolimbic pathway, the “reward pathway,” (Figure 1) is a dopaminergic pathway in the brain. The pathway connects the ventral tegmental area (VTA) in the midbrain to the ventral striatum (includes the nucleus accumbens (NAc) and the olfactory tubercle) of the basal ganglia in the forebrain. Release of dopamine (DA) (via signaling involving serotonin, endocannabinoids, enkephalin and GABA) from the mesolimbic pathway to the NAc regulates motivation and desire for reward stimuli. Drug and alcohol use can boost DA, a neurotransmitter that helps produce pleasurable feelings, thus promoting more cravings. As a person continues to abuse substances, the brain adapts by reducing the ability of cells in the reward circuit to respond to it. This reduces the euphoria that the person feels, inducing tolerance. Depletion of DA in this pathway, or lesions at its site of origin, or even serotonin depletion, decrease the extent to which an animal is willing to go to obtain a reward [7]. Brain adaptations often lead the person to become less able to derive pleasure from other things they once enjoyed (the thrill is gone). Continual long-term abuse of substances can cause changes to other brain chemical systems, affecting functions that include: learning, judgement, decision-making, and beahvior.

Following nearly three decades of genetic-based research related to identifying and characterizing addiction-related behavior, one of us (KB) coined the term “Reward Deficiency Syndrome.” Reward Deficiency Syndrome (RDS) is used to portray behaviors found to have gene-based association with hypo-dopaminergic function [8]. Among the literature that associate polymorphisms of reward genes with risk of RDS behaviors, the dopamine D2 receptor (DRD2) gene is one of the most widely studied as a receptor type. However, other genes are also involved, and it has been adequately established in association studies and animal research literature that, for example, polymorphisms of the serotonergic-2 A receptor and the catechol-O-methyltransferase genes pre-dispose individuals to aberrant RDS behaviors [9]. RDS, listed as a psychological disorder in the Sage Encyclopedia for Abnormal Psychology (2017), has gained wide acceptance in the scientific community, as a crucial factor in the etiology of all types of addictive, compulsive, and obsessive behaviors, like substance, and non-substance addictive behaviors, like gambling and gaming [10].

There are different strategies for managing addiction. One strategy is prevention of drug use. Principles of prevention of addiction advocates for early intervention (National Institute on Drug Abuse: Lessons from Prevention Research) and identifying risks at an early age [11]. Environmental factors and the induction of epigenetics can increase risk of addiction, so being aware what may be a risk factor for drug use may help individuals for prevention and in developing effective protective strategies [12].

Another strategy for managing addiction is to prevent relapse. People in recovery from substance use disorders are at an increase risk for relapse, even after years of not taking the substance. Relapse for substance use disorder is comparable to other chronic illnesses, such as hypertension and asthma [13]. In spite of over almost three decades of psychiatric genetic research with its 22,961 articles listed in Pubmed (12-1-19), and even after 17 years of addiction directed genetic research, Els [14] suggested that a number country’s public thinks of addiction as a moral failing rather than a medical condition, likely exacerbating the guilt and shame that recovering individuals experience.

### Rationale for GARS allelic selection

**Figure 2.**
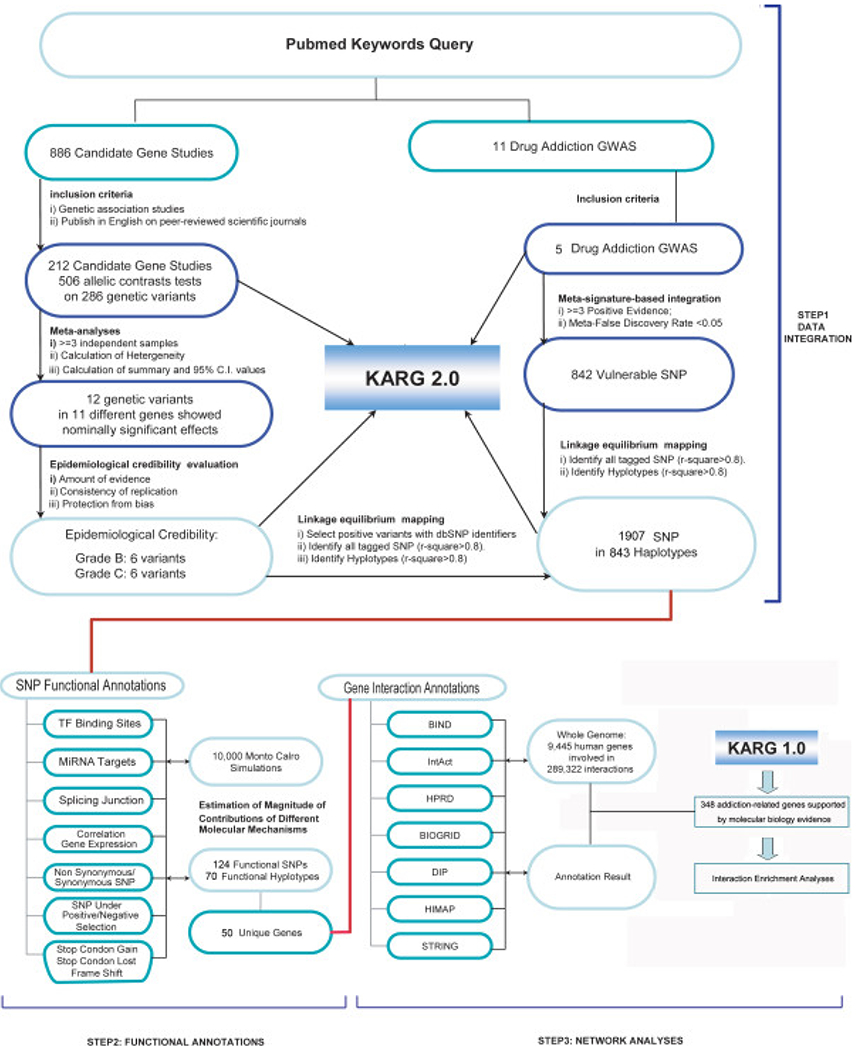
Pipelines for meta-analyses, functional SNP annotations and interaction analyses (with permission)

Since 1990 the field related to genetics of addiction has the state as of and Uhls’ group [15] provided a snapshot showing the state of the as of 2011, indicating the complex nature of genes linked to addictive behaviors particularly SUD. Figure 2 shows the overall pipeline of a meta-analyses of addiction-associated genetic variations, genome-wide analysis of the molecular mechanisms of implicated SNPs, and the pathways and gene interaction networks that might involve these genetic factors.

Meta-analyses of candidate gene association studies and GWAS were illustrated in detail in STEP 1. In total, 843 vulnerable haplotypes were identified, linked by 12 risk variants and 842 vulnerable SNPs. All data and knowledge were imported to an updated version of the knowledgebase for addiction-related genes (KARG 2.0, marked with a blue box). Haplotypes identified in STEP 1 were annotated with functional and regulatory elements (STEP 2). Taken from Interaction enrichment analyses between the susceptibility genes and addiction-regulated genes previously identified by molecular biology studies (KARG 1.0, marked with a blue box) were performed (STEP 3).

While the entire molecular biological community is interested in genetic risk for alcohol and substance addiction, and personalized medicine, presently, many are not aware of a genetic panel that demonstrates significant predictability to clinical risk. To this aim, we are highlighting this rather new and unique genetic test to provide this community an up-to date knowledge base. A Pubmed search for each gene represented in the GARS on 6-8-19 returned the following results (Table 1).

**Figure 3.**
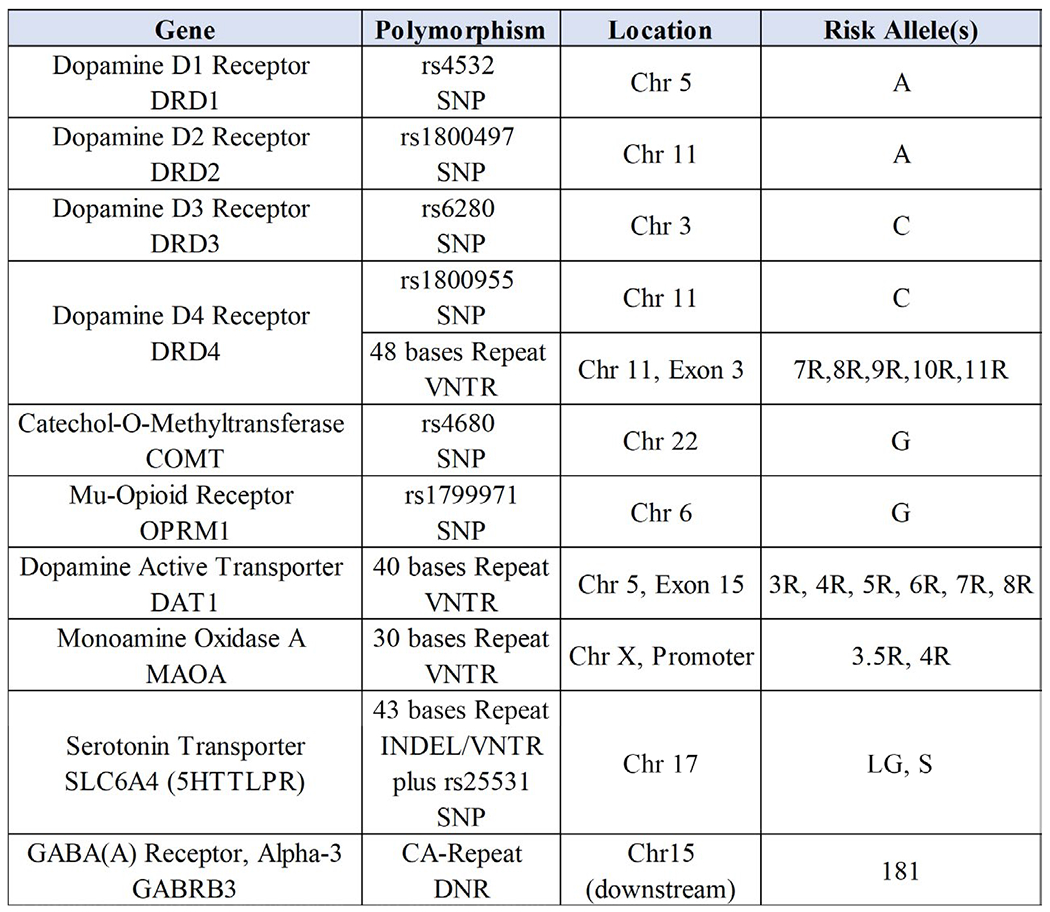
GARS panel

While it is true that no one to date has provided adequate RDS free controls there have been many studies using case –controls whereby SUD has been eliminated. We argue that this deficiency needs to be addressed in the field and if adopted appropriately many spurious results would be eliminated and as such it will reduce confusion providing a clearer understanding and an overhaul of the current state of the art of genetics underlining all addictive behaviors drug and non-drug or RDS. In Figure 3 we display the current polymorphic risk alleles of the GARS panel.

In addition we have highlighted a number of meta-analyses (if found) for each risk allele selected in GARS to point out a clear association compared to non-SUD controls and experimental SUD probands. Specifically, the genetic panel was selected for polymorphisms of a number of reward genes that have been correlated with chronic dopamine deficiency and drug related reward-seeking behavior.

An estimation based on these results herein, while not representative of all association studies known to date, of case- control studies provides significant associations whereby there are a total of **110,241** cases and **122,525** controls. A review of the related Figure 3 strongly suggest that while we may take argument concerning many of these so-called controls (e.g. blood donors) it is quite remarkable that there are a plethora of case –control studies indicating selective association of these risk alleles (measured in GARS) for the most part indicating a hypodopaminergia. Based on these results, we feel confident that albeit not having RDS free controls, there is sufficient evidence that each risk allele displayed in GARS relative to non-SUD controls associate as risk for prediction for drug and alcohol severity and dependence (Table 2).

Awareness of biological and environmental factors can impact a person’s risk of addiction. Scientists estimate that heritabilities of addictive disorders can range from 30-50% or possibly higher, depending on the substance [16]. Being cognizant of the biological risk may help individuals develop protective factors and help individuals see addiction as a medical condition. Additionally, standard tests, like the Addiction Severity Index [17] or Opioid Risk Tool [18], coupled with genetic polymorphic risk testing can help enhance understanding and achieve a personal medicine approach for each patient

## Methods and materials - Device description

The Genetic Addiction Risk Score (GARS) test is a non-diagnostic, DNA genetic testing tool. The Genetic Addiction Risk Score (GARS) is based on a qualitative genetic test for single nucleotide polymorphism detection of Substance Use Disoerder (SUD). We are detailing the methodology of the GARS test to provide the readerdhip with this important information and thereby reduce questions.

### Sample collection and processing utilized to obtain data

Buccal cells are collected from each patient using an established minimally invasive collection kit. Sterile Copan 4N6FLOQ Swabs (Regular Size Tip In 109MM Long Dry Tube with Active Drying System) were utilized for sample collection. Individuals collect cells from both cheeks by rubbing the swab at least 25 times on each side of their mouth, and then returned the swab to the specimen tube. For all steps of sample processing, appropriate controls including non-template controls and known DNA standards were included and verified [19].

An index of the genes included in the GARS panel and the specific risk polymorphisms are provided in Figures 4A–4C.

Each polymorphism was selected based on SUD a subset of Reward Deficiency Syndrome (RDS) and a known contribution to a state of low dopaminergic or hypodopaminergic functioning in the brain reward circuitry. Samples were also subject to sex determination using PCR amplification and capillary electrophoresis to detect *AMELX* and *AMELY* (*AMELX’s* intron 1 contains a 6 bp deletion relative to intron 1 of *AMELY*).

DNA was isolated from buccal samples using a Mag-Bind Swab DNA 96 Kit (Custom M6395-01, Omega Bio-Tek, Norcross, GA) with the MagMAX Express-96 Magnetic Particle Processor (Applied Biosystems, Foster City, CA). Extracted DNA was quantified for total human gDNA using the TaqMan RNaseP assay (Life Technologies, Carlsbad, CA) on a QuantStudio 12k Flex (Thermo Fischer Scientific, Waltham, MA).

Testing for genetic variation was performed using 1) Real-Time PCR with TaqMan® allele-specific probes on the QuantStudio 12K Flex, or 2) iPlex reagents on the Agena MassARRAY® system, plus 3) Proflex PCR and size separation using the SeqStudio Genetic Analyzer.

**Figure 5.**
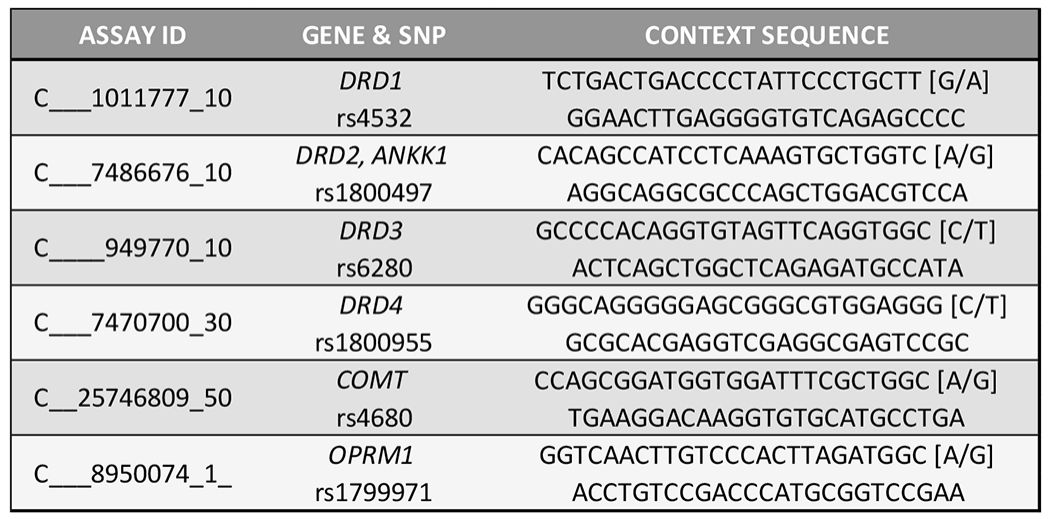
GARS single nucleotide polymorphism assays information

For genotyping the single nucleotide polymorphisms (Figure 4A) with Real-Time PCR with on the QuantStudio 12K Flex, commercially available or custom TaqMan RT-PCR assays (Thermo Fischer Scientific, Waltham, MA) were used (Figure 5 for Assay IDs and context sequences). For each reaction, 2.25 μL normalized DNA (10 ng total) was mixed with 2.75 μL assay master mix, and then subjected to RT-PCR amplification and detection. Manufacturer recommended thermal cycling conditions were utilized, and genotypes were called using TaqMan Genotyper Software v1.3 (Life Technologies, Carlsbad, CA).

For genotyping the single nucleotide polymorphisms with the Agena MassARRAY® system, iPlex reagents were used (Figure 4A for iPlex PCR primer sequences). Primers are multiplex, therefore only one reaction is required for each sample. For each reaction, 2 ul normalized DNA (10 ng total) was mixed with the iPlex Pro PCR cocktail. The reaction was amplified on a Pro Flex thermocycler with the Agena manufacturer PCR conditions. Amplified DNA was then SAP treated, followed by an extension. The iPLEX Extension Reaction Product was then desalted using a Dry Resin Method. Samples were then dispensed onto a 96 well SpectroCHIP Array using the MassARRAY Nanodispenser. Genotypes were called using the MassARRAY Analyzer Software.

**Figure 6.**
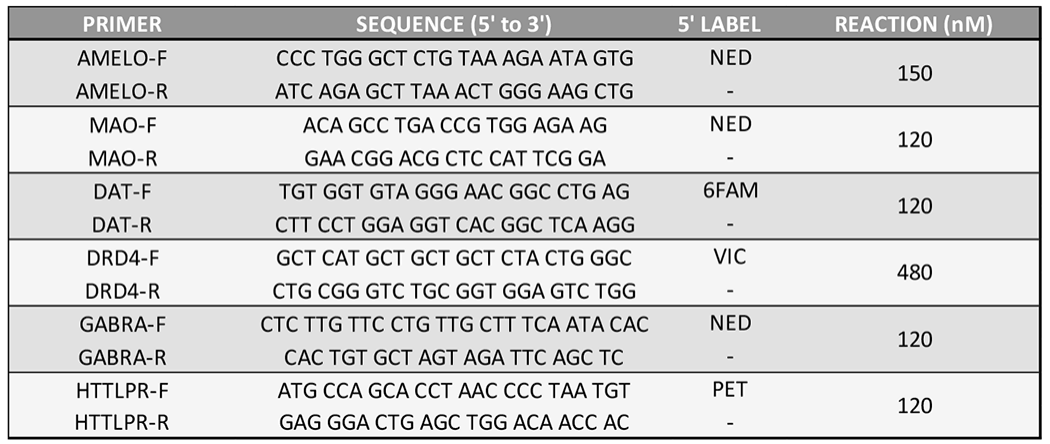
GARS repeats primer details

For fragment genotyping, two multiplexed PCR reactions (50 μL total volume) were required. Reaction A included 5’ fluorescently labeled primers forward primers and non-labeled reverse primers for *AMELOX/Y, DAT1, MAOA*, and the *GABRB3* dinucleotide repeat (with sets at 150 nM, 120 nM, 120 nM, and 480 nM primer concentrations, respectively). Reaction B included 5’ fluorescently labeled forward primers and non-labeled reverse primers for *DRD4* and the *SLC6A4* HTTLPR, all in 120 nM concentrations. For all PCR reactions, 2 ng DNA was amplified with primers, 25 μL OneTaq HotStart MasterMix (New England Biolabs, Ipswich, MA), and water. For reaction B, 5 μM 7-deaza-dGTP (Thermo Fischer Scientific, Waltham, MA) was added to the above recipe. Primers details are listed in Figure 6.

Amplifications were performed using a touchdown PCR method. An initial 95°C incubation for 10 min was followed by two cycles of 95°C for 30 s, 65°C for 30 s, and 72°C for 60 s. The annealing temperature was decreased every two cycles from 65°C to 55°C in 2°C increments (10 cycles total), followed by 30 cycles of 95°C for 30 s, 55°C for 30 s, and 72°C for 60 s, and a final 30-min incubation at 60°C, then hold at 4°C. A 10 μL aliquot of reaction B amplicon was further subjected to MspI restriction digest (37°C for 1 hr) to interrogate rs25531 (with 1U restriction enzyme and IX Tango Buffer, Thermo Fischer Scientific, Waltham, MA).

For fragment detection by capillary electrophoresis, reactions 1 and 2 were mixed in a 2:1 ratio. 1μL of this amplicon mixture was added to 9.5 μL mixed LIZ1200 size standard/formamide (Thermo Fischer Scientific, Waltham, MA recommended concentrations). For detection of rs25531, 1 uL of restriction digest mixture was added to 9.5 μL LIZ1200+formamide. Both mixtures were subjected to capillary electrophoresis on the SeqStudio (run time 60 min, voltage 5000 V, 10 sec injection at 1200 V) then analyzed with GeneMapper 5 software (Life Technologies, Carlsbad, CA). The patient/clinician who requests the test receives a personalized report discussing the results. The report provides a GARS Score (based on scale 1-22) that is the sum of all risk alleles for that individual. Various substance and non-substance behaviors are listed as high, moderate, or low risk behavior frequency for that individual. The reports are designed to help users understand the meaning of their results and any appropriate actions that may be taken.

## Summary of initial GARS study

We are briefly summarizing herein the first study of an association between the Genetic Addiction Risk Score (GARS) and the Addiction Severity Index -Media Version (ASI-MV) among patients from treatment facilities (submitted for publication).

The initial sample of 393 subjects who provided saliva for genotyping, was drawn, from eight geographically diverse treatment centers in the United States. The available sample size of 273 (69%) consisted of individuals who had also completed the ASI-MV questionnaire [17]. The alcohol, and drug severity scores in the ASI-MV were determined using a proprietary algorithm developed by Inflexxion. A laboratory located at the Institute for Behavioral Genetics (University of Colorado Boulder) performed standard genotyping for specific polymorphic risk alleles derived from a panel of reward genes. The subjects, participating in the pilot phase of the GARS analysis self-reported their race as White at 88.1% (n = 244) and were 57.8% (n = 160) male. The average age of the of subjects was 35.3 years (S.D. =13.1, maximum age = 70, minimum age = 18). This study is a statistical analysis that compared a number of risk alleles to the ASI-MV alcohol and drug severity score of each subject.

Among the ASI analysis sample the number of risk alleles detected ranged from 3 to 15, and the average was 7.97 (S.D. = 2.34) with a median of 8.0. Preliminary examination of the relationship between GARS genotype panel and the Alcohol Risk Severity Score using the Fishers Exact Test revealed a significant predictive relationship (X^2^ = 8.84, df = 1, p = 0.004 2 tailed) which remained significant after controlling for age [Hardy-Weinberg Equilibrium intact]. Both age and genetic addiction risk scores were predictive of higher alcohol severity scores as assessed with the ASI-MV. In fact, a lower ASI-score predicted a lower GARS score. To account for non-normality in the distribution, drug scores were transformed to (Log_10_) before analysis of the relationship between the GARS panel and ASI-MV Drugs Risk Severity Score. The relationship between the GARS panel and the Drug Risk Severity Score was found to be similar but less robust than the observation for the Alcohol Risk Severity. Preliminary examination revealed a nominally significant relationship (B = −0.122, t = −1.91, p = 0.057 −2 tailed) in this study, following apriori hypothesis of an association of GARS and ASI predictability of risk in which a one-tailed analysis revealed (P=0.028) for the drug severity. The predictive value of GARS was more robust for alcohol risk severity (a score equal or greater that 7) and for drug risk severity (a score equal or greater that 4). A limitation of this study relates to the attempt of matching an objective score (genes) with a score from a subjective self-report (ASI).

These results show the GARS test to be a useful predictor of susceptibility to problematic substance use, especially alcoholism. In future studies using highly screened cohorts eliminating all Reward Deficiency Syndrome (RDS) behaviors, TOD scores will be analyzed for each risk allele to determine weighted associations that could lead to even more accurate predictability of the GARS test.

### Population GARS prevalence

PubMed provides frequency data of major and minor allele, but not population prevalence. SNPedia provides population diversity percentages for homozygous SNP; homozygous normal; and heterozygous for all but one of our SNPs, in the following populations (rs4532; rs1800497; rs6280; rs1800955; rs4680 and rs1799971). Unfortunately, currently, there is no population prevalence data on variable number tandem repeats or dinucleotide repeats (Tables 3 and 4).

### Sampling of data retrieval

Tables 4 and 5 illustrates the utility of the GARS across various ethnic groups in a population of 293. We are presenting this data as a sampling of types of data collection that could be obtained utilizing the above cited techniques. The prevalence in either N or % of each risk allele is displayed in Tables 4 and 5 including: **COMT, DRD1, DRD2, DRD3, DRD4, OPM1, DATl-repeats, MAOA, GABRB3, DRD4-repeats and HTTLPR.**

## Future perspectives

These data and other analyses of GARS have allowed for the current utilization of precise genetic guided therapy coined “Precision Addiction Management” (PAM®) [20]. Simply, “Precision Addiction Management” (PAM®) uses the GARS to customize KB220PAM [21] formulations to deliver putative dopamine homeostasis based on developed algorithms matched to polymorphic results. To date there have been 42 published studies on KB220 related to many RDS behaviors [22]. There is evidence derived from animal and human studies using BOED neuroimaging and behavioral methodologies that support homeostatic activation of brain dopamine in the reward circuitry by KB220 variants, as well as anti-substance seeking and modification of RDS behaviors [23–28]. RDS encompasses behaviors like PTSD, ADHD, over-eating, shopping, hoarding and related RDS cognitive insults. Combating the drug crisis requires PBM across ethnic groups, to induce dopamine homeostasis to those born with RDS predisposition [29].

Previously Blum developed a RDS inventory (questionnaire) which has been significantly modified by Demetrovics & Blum to display 29 items. In other work conducted by Demetrovics’ group involved in the PGA study, a wide spectrum national study was carried out on approximately 1500 adolescents and young adults. These data will be analyzed for 1) explore the characteristics ofRDS addictions; 2) Analyze the relationship between both drug and non-drug addictive behaviors; 3) explore the possible genetic markers for all types ofRDS behaviors; 4) provide a genetic map using GARS for RDS type addictive behaviors; 5) explore the possible distinct & overlapping common psychological and genetic characteristics of different types of substance use and behavioral addictions and 6) Provide a multidisciplinary approach and test and test possible psychological and genetic interaction effects; 7) develop modifications to the current GARS if necessary based on forthcoming analytic results.

It is agreed that both specific psychological and genetics may play an important role in the development of addiction. For example, studies have indicated that personality traits (e.g. schizoid/avoidance; sensation seeking and impulsivity) are associated with SUD [30], Barnes et al. [31]; Saramon et al. [32] and behavioral addictions [ 33]. However, the exact mechanisms of how these traits are risk factors of addictions are unknown. To address this conundrum Blum’s group over a 50 year sojourn confirmed in several studies the concept of a genetically induced hypodopaminergic trait leasing to reward deficiency syndrome that in turn underlies impulsive and addictive behaviors [34–38]. According to Comings and Blum [38] biogenic model, specific genetic variants can cause dysfunctions in the brain reward cascade [39] evoking a hypodopaminergia. The take home message is that the hypodopaminergic brain requires a “dopamine fix” to feel good to subsequently lead to RDS seeking behaviors. With this stated research directed as suggested by the PGA study, could widen the horizon of addiction like phenotypes both drug and non-drug with novel associations that might lead to new perspectives and assist in the identification of yet unknown correlates of these RDS behaviors.

**Figure 7.**
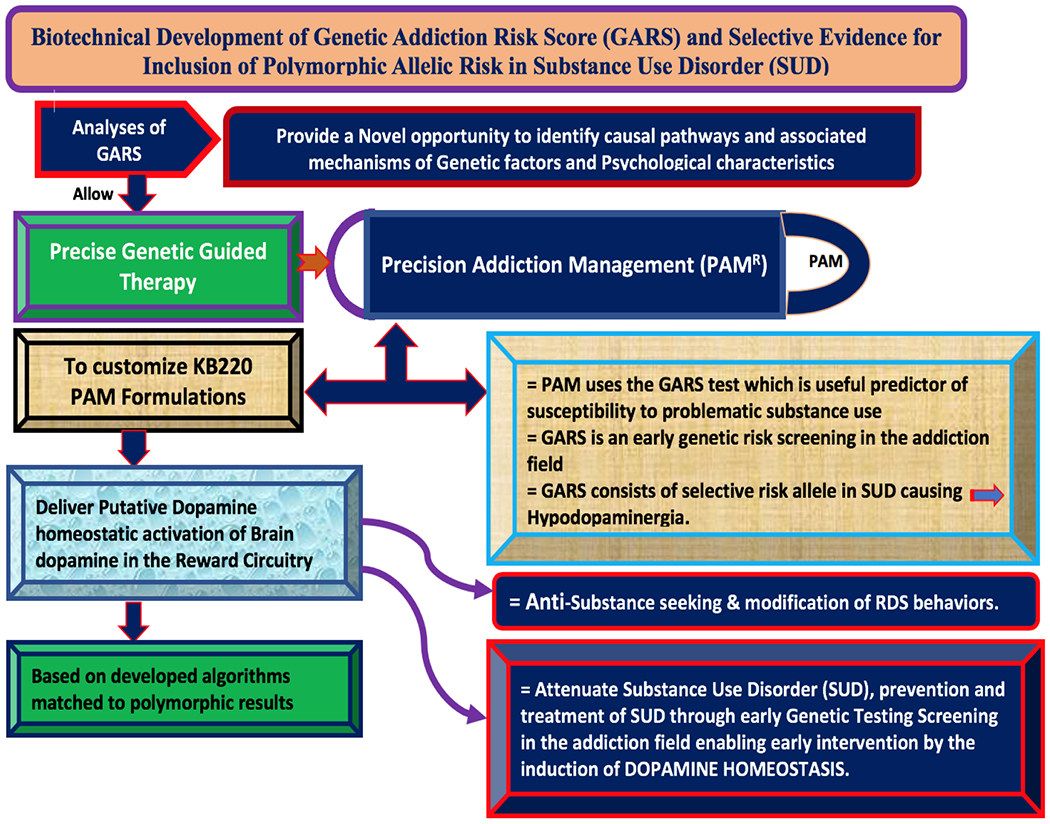
Provides a simple schematic portraying our proposal

In order to provide a simple schematic portraying our proposal (Figure 7).

## Summary

It is the goal through this novel model that by using PBM the addiction field will have a synergistic tool along with MAT or even alone, to overcome dopamine dysregulation either surfeit (adolescents) or deficit (adults) by the induction of “ dopamine homeostasis” to help attenuate SUD by enabling early intervention through genetic testing [23,24].

## Figures and Tables

**Figure 4A.**
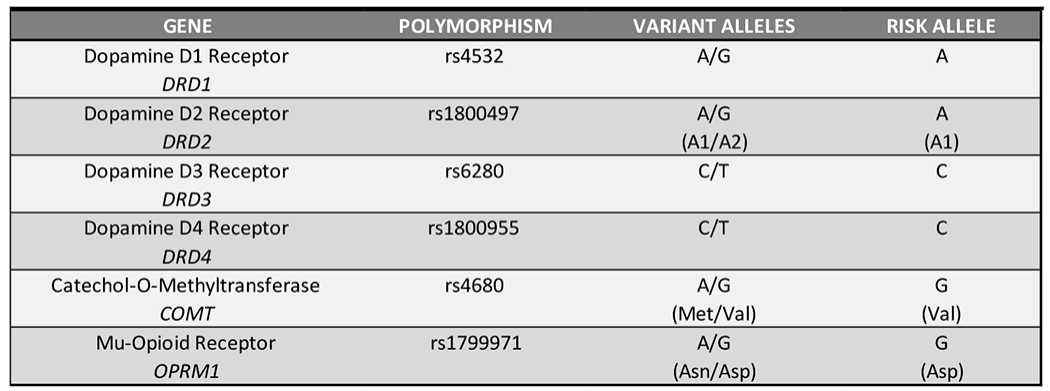
Single nucleotide polymorphisms (SNPs)

**Figure 4B.**
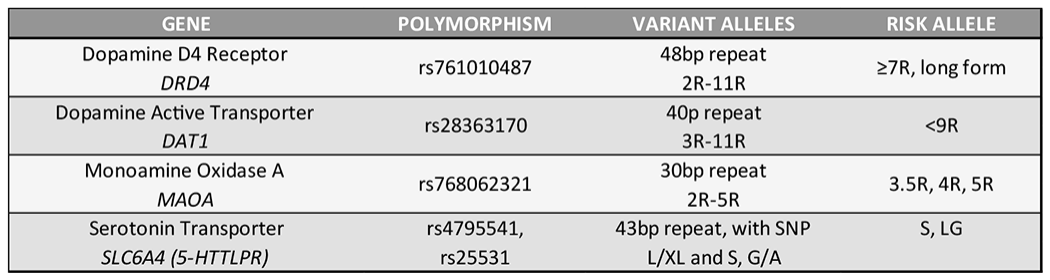
Simple sequence repeats (Variable number tandem repeats and insertion/deletions)

**Figure 4C.**
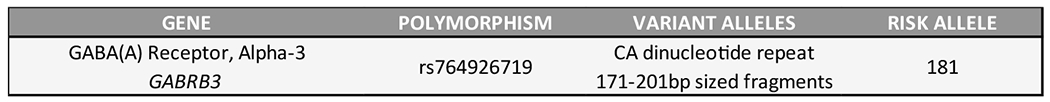
Dinucleotide repeats

**Table 1.** Population number of PUBMED listed studies per gene as of 6-12-19

Name	PubMed Listed Protein	PubMed Listed Gene
SEROTONIN TRANSPORTER	10,146	4,532
COMT	4,879	2,785
MONAMINE OXIDASE – A	23,063	1,899
DOPAMINE D1 RECEPTOR	12,249	1,791
DOPAMINE D2 RECEPTOR	23,020	4,724
DOPAMINE D3 RECEPTOR	4,037	857
DOPAMINE D4 RECEPTOR	2499	1,294
DOPAMINE TRANSPORTER	10,586	2,830
OPIOID MU RECEPTOR	13,854	1,873
GABA RECEPTOR(s)	29,004	3,745
TOTAL	133,337	26,330

**Table 2.** Represents the GARS Polymorphisms and only positive case –control association and longitudinal studies

Gene	Poly-morphism	Association	Case-Control	Reference
**DRD1**	Rs4532	Rs 4532 could predict decreased postopioid dependence pleasure.	The present study included N=425 unrelated opioid addicts registered in the Methadone Maintenance Treatment Program at Xi’an Mental Health Center of China. The OD diagnosis was established using DSM-IV criteria and based on medical record, urine test, and interview. The controls consisted of N=514 unrelated healthy persons who had never been diagnosed with substance abuse and mental illness.	Zhu F (2013) Dopamine D1 receptor gene variation modulates opioid dependence risk by affecting transition to addiction. *PloS one 8:* e70805.
	Rs4532	Statistically significant associations of polymorphisms in DRD1 rs4532 with alcoholism were found.	In another study N=140 male alcohol dependent subjects attending the outpatient department (OPD) at National Drug Dependence Treatment Centre, AIIMS, were screened. A total of N=122 unrelated healthy male employees of the hospital, without any history of substance use (except nicotine) were included as controls in the study.	Prasad P (2013). Case-control association analysis of dopamine receptor polymorphisms in alcohol dependence: a pilot study in Indian males. BMC research notes, 6, 418. doi:10.1186/1756-0500-6-418
	Rs4532	A specific haplotype rs686*T-rs4532*G within the DRD1 gene was significantly more precisely associated with alcohol dependence in our sample (p = 5 x 10(−6)).	A third study analyzed a population of 134 patients with alcohol dependence, also assessing more homogeneous (severe) phenotypes, comparing this sample with a healthy control population, assessing two SNPs within the DRD1 gene in order to depict the role of DRD1 polymorphisms and haplotypes	Batel P, Houchi H, Daoust M, Ramoz N, Naassila M, Gorwood P. A haplotype of the DRD1 gene is associated with alcohol dependence. Alcohol Clin Exp Res. 2008 Apr;32(4):567-72. doi: 10.1111/j.1530-0277.2008.00618.x.
**DRD2**	Rs1800497	DRD2/ANKK1 TaqIA polymorphism was significantly associated with increased risk of opioid dependence under homo zygote, dominant, and recessive genetic model, respectively (homozygote: OR=1.546, 95%CI=1.279-1.87; dominant: OR=1.265, 95%CI=1.055-1.516; recessive: OR=1.409, 95%CI=1.182-1.680).	A total of 25 available case-control studies testing the association between the polymorphism and common illicit drug dependence were examined through Oct 2013. Pooled odds ratios (ORs) and 95% confidence intervals (CI) were estimated using fixed- and random-effects models when appropriate.	Deng XD (2015) Association between DRD2/ANKK1 TaqIA polymorphism and common illicit drug dependence: evidence from a **meta-analysis**. Hum Immunol. 2015 Jan;76(1):42-51. doi:10.1016/j.humimm.2014.12.005.
	Rs1800497	TaqI A1 polymorphism was significantly increased for opioid dependence risk (homozygote comparison: OR, 2.06; 95% CI, 1.25-3.42; dominant comparison: OR, 1.34; 95% CI, 1.08-1.67).	A meta-analysis, including N=6,846 opioid dependence cases and N=4,187 controls from 22 individual studies	Chen D, Liu F, Shang Q, Song X, Miao X, Wang Z. Association between polymorphisms of DRD2 and DRD4 and opioid dependence: evidence from the current studies. Am J Med Genet B Neuropsychiatr Genet. 2011 ;156B(6):661-70. doi: 10.1002/ajmg.b.31208.
	Rs1800497	rs1800497 was significantly associated in men with excessive alcohol consumption.	The study sample (N = 1533, of which 746 were women) consisted of 653 heavy consumers and N= 880 very low consumers from the Spanish sub-cohort of the European Prospective Investigation into Cancer and Nutrition (EPIC) cohort.	Celorrio D, Munoz X, Amiano P, Dorronsoro M, Bujanda L, Sánchez MJ, Molina-Montes E, Navarro C, Chirlaque MD, MariaHuerta J, Ardanaz E, Barricarte A, Rodriguez L, Duell EJ, Hijona E· Herreros-Villanueva M,· Sala N Alfonso-Sanchez MA,de Pancorbo MM. Influence of Dopaminergic System Genetic bâriation and Lifestyle Factors on Excessive Alcohol Consumption. Alcohol Alcohol. 2016 May;51 (3):258-67. doi: 10.1093/alcalc/agv114.
	Rs1800497	Genetic variation in rs1800497 play an equal role in predicting alcohol drinking two years later and are most important in predicting the increase in alcohol consumption	Examined N= 736 adolescents from the IMAGEN **longitudinal study** for alcohol drinking during early (mean age= 14.37) and again later (mean age=16.45) adolescence.	Heinrich A, IMAGEN consortium. Prediction of alcohol drinking in adolescents: Personality-traits, behavior, brain responses, and genetic variations in the context of reward sensitivity. Biol Psychol. 2016 ;118:79-87. doi: 10.1016/j.biopsycho.2016.05.002.
	Rs1800497	Rs1800497 genotype distribution is significantly associated to Alcohol Dependence (O.R.=1.551, p=0.023), with A1 * allele displaying an O.R.=1.403 (p=0.029).	The study design was a case-control. In total, N=−280 alcoholic subjects (213 men and 67 woman) and N=280 age- and sex-matched control subjects were recruited for this study	Vlignini F, Napolioni V, Codazzo C, Carpi FM, Vitali M, Romeo M, Ceccanti M. DRD2/ANKK1 TaqIA and SLC6A3 VNTR polymorphisms in alcohol dependence: association and gene-gene interaction study in a population of Central Italy. Neurosci Lett. 2012;;522(2):103-7. doi: 10.1016/j.neulet.2012.06.008.
	Rs1800497	Homozygous genotype variants (A1A11 A2A2) were over-represented in case groups compared to controls, while heterozygotes were more frequently observed in control group, but no statistically significant difference was found. Analysis of genotype A1A1 frequency showed 6% and 2% in case and control groups respectively. Statistical analysis pointed statistically significant overrepresentation of A1A1 genotype in case group, as opposed to expected –normal distribution with controls (OR=1.532 (CI=1.001-2.344) i *p*<0.05. This implies that A1A1 carriers have 1.5 fold increased risk for development of heroin addiction with 30-40% lower D2 receptors	100 patients on Metadone Maintenance Treatment (MMT) and 100 age and sex matched healthy volunteers	Mehić-Basara, N, Oruč, L, Kapur-Pojskić, L, Ramić, J. (2013). Association of dopamine receptor gene polymorphism and psychological personality traits in liability for opioid addiction. *Bosnian journal of basic medical sciences, 13(3)*, 158-162. doi: 10.17305/bjbms.2013.2355
	Rs1800497	In single marker analysis the TaqIA (rs1800497) and TaqIB (rs1079597) variants were associated with heroin dependence	303 heroin dependent subjects and 555 healthy controls were genotyped for Rs1800497 and TaqIB (rs1079597)	Vereczkei A, Demetrovics Z, Szekely A, Sarkozy P, Antal P, Szilagyi A, Sasvari-Szekely M, Barta C. Multivariate analysis of dopaminergic gene variants as risk factors of heroin dependence. *PLoS One.* 2013 Jun 28;8(6):e66592. doi: 10.1371/joumal.pone.0066592. PubMed PMID: 23840506; PubMed Central PMCID: PMC3696122.
	Rs1800497	A random/fixed-effects meta-analyses using allelic contrasts found that the TaqIA (Rs1800497) significantly associated with addiction at < 0.0001	A meta-analytic comparison of single marker analysis of the TaqIA (rs1800497) ,whereas A2 was compared to A1 in 6312 Cases vs. 7424 Controls in 20 independent studies	Li CY, Zhou WZ, Zhang PW, Johnson C, Wei L, Uhl GR. Meta-analysis and genome-wide interpretation of genetic susceptibility to drug addiction. *BMC Genomics.* 2011 Oct 15; 12:508. doi: 10.1186/1471-2164-12-508. PubMed PMID: 21999673; PubMed Central PMCID: PMC3215751.
	Rs1800497	Meta-analysis demonstrated both allelic and genotypic association between the TaqIA polymorphism and AD susceptibility [allelic: *P(Z)*=1.1×10^−5^, OR=1.19; genotypic: *P(Z)*=3.2×10^−5^, OR=1.24]. The association remained significant after adjustment for publication bias using the trim and fill method	A large-scale meta-analysis to confirm the association between the TaqIA polymorphism and the risk for AD in over 18,000 subjects included in 61 case-control studies that were published up to August 2012	Wang F, Simen A, Arias A, Lu QW, Zhang H. A large-scale meta-analysis of the association between the ANKK1/DRD2 TaqIA polymorphism and alcohol dependence. *Hum Genet.* 2013 Mar; 132(3):347-58. doi: 10.1007/s00439-012-1251-6. Epub 2012 Dec 1. PubMed PMID: 23203481; PubMed Central PMCID: PMC3570676.
**DRD3**	DRD3 Ser9Gly polymorphism (rs6280)	Linear regression analysis controlling for age, sex, diagnosis, and self-reported anhedonia indicated that during receipt of unpredictable monetary reward the glycine allele was associated with a greater reduction in D2/3 receptor binding (i.e. increased reward-related DA release) in the middle caudate (r^2^ explained=0.24, β-weight=0.5, t=3.3, p = 0.003) and the ventral striatum	Twenty-six right-handed HCs (15 women; mean age 34.4±8.3) and 10 currently depressed, unmedicated patients with MDD (8 women; mean age 38.2±11.3) participated in the study	Savitz J, Hodgkinson CA, Mlartin-Soelch C, et al, The functional DRD3 Ser9Gly polymorphism (rs6280) is pleiotropic, affecting reward as well as movement. PLoS one, 8:e54108, (2013).
	rs6280	This SNP was significantly associated with both AD overall (χ(2) = 10.09 and p = 0.001, and χ(2) = 10.60 and p = 0.005, for the recessive and additive models, respectively) and with Lesch type I AD (χ(2) = 11.70 and p = 0.001, and χ(2) = 11.70 and p = 0.003, for the recessive and additive models, respectively). The findings of this study suggest that the DRD3 rs6280 polymorphism is associated with the development of both AD overall and Lesch type I AD	The DRD3 rs6280 SNP was genotyped in a case-control sample comprising 245 AD patients and 130 healthy controls (HCs). Alcohol Use Disorders Identification Test (AUDIT) scores were also compared relative to genotype in all of the participants.	Kang SG, Lee BH, Lee JS, Chai YG, Ko KP, Lee HJ, Han DMI, Ji H, Jang GH, Shin HE. DRD3 gene rs6280 polymorphism may be associated with alcohol dependence overall and with Lesch type I alcohol dependence in Koreans. Neuropsychobiology. 2014;69(3):140-6. doi: 10.1159/000358062.
	rs6280	The rs6280 and rs9825563 variants showed association with the development of early-onset heroin dependence. Authors suggest DRD3 is possibly a genetic factor in the development of early-onset heroin dependence	Eight polymorphisms in DRD3 were analyzed in 1067 unrelated Han Chinese subjects (N=566 heroin dependence patients and N= 501 controls). All participants were screened using the same assessment tool and all patients met the criteria for heroin dependence	Kuo SC, Yeh YW, Chen CY, Huang CC, Chang HA, Yen CH, Ho PS, Liang CS, Chou HW, Lu RB, Huang SY. DRD3 variation associates with early-onset heroin dependence, but not specific personality traits. Prog Neuropsychopharmacol Biol Psychiatry. 2014 Jun 3;51 :l-8. doi: 10.1016/j.pnpbp.2013.12.018.
	rs6280	ATA haplotype combination for SNPs rs324029, rs6280, and rs9825563, respectively, was significantly associated with total amphetamine dependence patients (p = 0.0003 after 10,000 permutations).	A total of 1060 unrelated Han Chinese subjects (N=559 AD patients and N=501 controls) were screened using the same assessment tool and genotyped for eight DRD3 polymorphisms.	Kuo SC, Yeh YW, Chen CY, Huang CC, Chen TY, Yen CH, Liang CS, Ho PS, Lu RB, Huang S Y. Novelty seeking mediates the effect of DRD3 variation on onset age of amphetamine dependence in Han Chinese population. Eur Arch Psychiatry Clin Neurosci. 2018 Apr;268(3):249-260. doi: 10.1007/s00406-016-0754-x.
		Single-marker analyses provided nominally significant evidence for associations of rs6280 in both heroin and cocaine addiction.	This study examined the association of 98 single nucleotide polymorphisms in 13 dopamine-related genes with heroin addiction (OD) and/or cocaine addiction (CD) in a sample of N= 801 African Americans (315 subjects with OD ± CD, 279 subjects with CD, and 207 controls).	Levran O, Randesi MI, da Rosa JC, Ott J, Rotrosen J, Adelson MI, Kreek MIJ. Overlapping dopaminergic pathway genetic susceptibility to heroin and cocaine addictions in African Americans. *Ann Hum Genet.* 2015 May;79(3):188-98. doi: 10.1111/ahg.12104. Epub 2015 Feb 27. PubMed PMID: 25875614; PubMed Central PMCID: PMC4399004.
**DRD4**	Rs180095 48bP repeat VNTR	521 C/T SNP (rs1800955) of the DRD4 gene showed nominal association with a possible protective effect of the C allele for heroin dependence	N-303 heroin dependent subjects and N=555 healthy controls were genotyped for Rs1800955, rs936462 and rs747302 of the DRD4 gene.	Vereczkei A, Demetrovics Z, Szekely A, Sarkozy P, Antal P, Szilagyi A, Sasvari-Szekely M, Barta C. Multivariate analysis of dopaminergic gene variants as risk factors of heroin dependence. *PLoS One.* 2013 Jun 28;8(6):e66592. doi: 10.1371/joumal.pone.0066592. PubMed PMID: 23840506; PubMed Central PMCID: PMC3696122.
		The *DRD4* polymorphism 120 bp tandem duplication, located 1.2 kb upstream of the initiation codon was analyzed. Statistically significant associations of polymorphisms in in *DRD4* with alcoholism were found.	A total of 90 cases (alcohol dependent males) and 122 age and ethnicity matched healthy male controls were recruited in the study by following DSM-IV criteria.	Prasad P, Ambekar A, Vaswani M. Case-control association analysis of dopamine receptor polymorphisms in alcohol dependence: a pilot study in Indian males. *BMC Res Notes.* 2013 Oct 17;6:418. doi: 10.1186/1756-0500-6-418. PubMed PMID: 24135011; PubMed Central PMCID: PMC3 853477.
	Rs180095	The purpose of this longitudinal study was to investigate a genetic moderation effect of dopamine receptor-4 gene (*DRD4*) alleles that have 7 or more repeats on the efficacy of a preventive intervention to deter rural African American adolescents’ substance abuse In the so called control group the AA that carried at least one copy of the DRD4 7 repeat initiated drinking, behavior as a risk.	Adolescents (*N* = 502, *M* age = 16 years) were assigned randomly to the Strong African American Families–Teen (SAAF-T) program or to a control condition and were followed for 22 months.	Brody GH, Chen YF, Beach SR, Kogan SM, Yu T, Diclemente RJ, Wingood GM, Windle M, Philibert RA. Differential sensitivity to prevention programming: a dopaminergic polymorphism-enhanced prevention effect on protective parenting and adolescent substance use. *Health Psychol.* 2014 Feb;33(2):182-91. doi: 10.1037/a0031253. Epub 2013 Feb 4. PubMed PMID: 23379386; PubMed Central PMCID: PMC3695005.
		A random/fixed-effects meta-analyses using allelic contrasts found that the DRD4 48 BP repeat λ/NTR associated with addiction at < 0.06)	A meta-analytic comparison of single marker analysis of the DRD4 48 BP repeat VNTR was compared 2324 cases vs. 1932 controls in 6 independent studies	Li CY, Zhou WZ, Zhang PW, Johnson C, Wei L, Uhl GR. Meta-analysis and genome-wide interpretation of genetic susceptibility to drug addiction. *BMC Genomics.* 2011 Oct 15; 12:508. doi: 10.1186/1471-2164-12-508. PubMed PMID: 21999673; PubMed Central PMCID: PMC3215751.
	Rs180095	This longitudinal study found Alcohol use at age 33 was predicted by previous friends’ alcohol use and correlated with current friends’ alcohol use only for carriers of the DRD4 long allele. The long allele of DRD4 is associated with increased susceptibility to peer influences on alcohol use in young adulthood	Participants (N=340; 59% female; 98% White) reported on their own and their friends’ alcohol use at four time points between mean ages 17 and 33. Multigroup modeling tested differences in model paths and covariances across high vs. low risk DRD4 polymorphisms.	Mrug S, Windle M. DRD4 and susceptibility to peer influence on alcohol use from adolescence to adulthood. *Drug Alcohol Depend.* 2014 Dec 1; 145:168-73. doi: 10.1016/j.drugalcdep.2014.10.009. Epub 2014 Oct 23. PubMed PMID: 25457740; PubMed Central PMCID: PMC4268151.
	Rs180095	In this study N=50 carried DRD4 7R and N=91 did not carry the DRD4 7 R. A significant gene-by-environment interaction suggested that the same-day association between witnessing substance use and antisocial behavior was significantly stronger among adolescents with, versus without, with the *DRD4*-7R allele. Providing evidence for risk for SUD.	Young adolescents (N=151) at heightened risk for both exposure to substance use. Adolescents were, on average, 13 years of age. *DRD4* genotype was determined. They split adolescents into two groups: (1) those who possessed at least one copy of the 7R allele on either chromosome versus (2) those who did not possess a copy of the 7R allele on either chromosome.	Russell MA, Wang L, Odgers CL. Witnessing substance use increases same-day antisocial behavior among at-risk adolescents: Gene-environment interaction in a 30-day ecological momentary assessment study. *Dev Psychopathol.* 2016 Nov;28(4pt2):1441-1456. doi: 10.1017/S0954579415001182. Epub 2015 Dec 9. PubMed PMID: 26648004; PubMed Central PMCID: PMC5330259.
	Rs180095	DRD4 7R more commonly found in individuals with high quantity/frequency of drug use compared with controls	The DRD4 48 BP repeat (7R) was evaluated in 184 substance abusers and 122 controls	Vandenbergh DJ,Rodriguez LA,Hivert E,Schiller JH,Villareal G,Pugh EW,Lachman H,Uhl GR. Long forms of the dopamine receptor (DRD4) gene VNTR are more prevalent in substance abusers: no interaction with functional alleles of the catechol-o-methyltransferase (COMT) gene. Am J Med Genet. 2000 Oct 9;96(5):678-83.
	Rs180095	DSM IV methamphetamine disorders was assessed in each subject. 7-repeat allele more commonly found in methamphetamine abusers than controls	N=416 methamphetamine users and N=435 controls genotyped for presence vs. absence of 7- repeat allele	Chen CK,Hu X,Lin SK,Sham PC,Loh el-W,Li T,Murray RM.Ball DM. Association analysis of dopamine D2-like receptor genes and methamphetamine abuse. Psychiatr Genet. 2004 Dec;14(4):223-6.
	Using individual alleles and grouping 5-7 repeats as “long”	Association of 7-repeat allele and alcohol dependence in case control design	Case control design-218 cases and 197 controls, −76 alcoholics and their parents	Franke P, Wang T, Nothen MM, Knapp M, Neith H, Lichtermann D, Meyer Zur Caprllen K, Sander T, Propping P, Maier W. DRD4 exon III VNTR polymorphism: a case-control and family-based association approach. Addiction Biology. 2000 Jan;5(1):289–95.
	Rs180095	DSM-IV opioid dependence. Association of 7-repeat allele and opioid dependence	141 opioid dependent patients and 110 controls	Kotler M, Cohen H, Segman R, Gritsenko I, Nemanov L, Lerer B, Kramer I, Zer-Zion M, Kletz I, Ebstein RP. Excess dopamine D4 receptor (D4DR) exon III seven repeat allele in opioid-dependent subjects. Mol Psychiatry. 1997 May;2(3):251–4.
	DRD4 haplotype (including exon 3 VNTR)	Association of DRD4 haplotype (including exon 3 VNTR) with methamphetamine abuse and interaction of exon 3 VNTR with COMT 158 Val/Met polymorphism	228 methamphetamine abusers and 181 controls	Li T, Chen CK, Hu X, Ball D, Lin SK, Chen W, Sham PC, Loh el-W, Murray RM, Collier DA. Association analysis of the DRD4 and COMT genes in methamphetamine abuse. Am J Med Genet B Neuropsychiatr Genet. 2004 Aug 15;129B(1):120–4.
	DRD4 5 repeat allele	DSM-III-R alcohol dependence Increased frequency of DRD4 5 repeat allele in alcoholics with protective ALDH2 allele compared with controls on alcoholics without this protective	655 Japanese alcoholics and 144 unrelated controls	Muramatsu T, Higuchi S, Murayama M, Matsushita S, Hayashida M. Association between alcoholism and the dopamine D4 receptor gene. J Med Genet. 1996 Feb;33(2):113–5.
	4 repeat homozygotes compared with all others	DSM-IV alcohol dependence in parents. 4-repeat homozygotes less common in children of alcoholics	18 children of alcoholics and 23 children of nonalcoholic parents	Namkoong K, Cheon KA, Kim JW, Jun JY, Lee JY. Association study of dopamine D2, D4 receptor gene, GABAA receptor beta subunit gene, serotonin transporter gene polymorphism with children of alcoholics in Korea: a preliminary study. Alcohol. 2008 Mar;42(2):77–81
	Rs180095 48bP repeat VNTR	The long-repeat alleles of the DRD4 exon 3 VNTR polymorphism were found more frequently in the heroin addicts (P=0.019).	N=894 male heroin addicts without other psychiatric disorders, were recruited as subjects. Another community N=180 males were selected randomly as controls.	Chien CC, Lin CH, Chang YY, Lung FW. Association of VNTR polymorphisms in the MAOA promoter and DRD4 exon 3 with heroin dependence in male Chinese addicts. World J Biol Psychiatry. 2010 Mar;11(2 Pt2):409-16. doi: 10.3109/15622970903304459.
**DAT1**	base repeat VNTR	This analysis showed that the 6R6R genotype was associated with crack cocaine addiction (OR = 1.844; CI = 1.101-3.089; p = 0.020).	Compared allele and genotype frequencies of a 30-bp variable number of tandem repeats (VNTR) polymorphism of the DAT1 gene, located at intron 8, between adult crack cocaine users and non-addicted individuals. A cross-sectional sample of N=239 current adult crack abusers or dependents from in- and outpatient clinics and N= 211 control individuals was collected in Brazil.	Stolf AR, Muller D, Schuch JB, Akutagava-Martins GC, Guimaraes LSP, Szobot CM, Halpern R, Kessler FHP, Pechansky F, Roman T^2^. Association between the Intron 8 VNTR Polymorphism of the DAT1 Gene and Crack Cocaine Addiction. Neuropsychobiology. 2017;75(3):141-144. doi: 10.1159/000485128.
	9R allele compared to 10R.	Variants in DAT VNTR, showed that the presence of the 9-9 genotype significantly increases the risk of irritability and direct aggressiveness more than six and 10 times with respect to the 9-10 genotype in heroin addicts compared to controls. 9-repeat allele of the DAT polymorphism confers increased susceptibility to antisocial - violent behavior and aggressiveness in heroin addicts.	Polymorphism of a variable number of tandem repeats (VNTR) in the 3’ untranslated region of exon 15 of the SLC6A3 gene, coding for the dopamine transporter (DAT). The repeat number of the DAT polymorphism was assessed in N=125 healthy subjects and N= 104 heroin-dependent subjects (including 52 addicted individuals with violent behavior and criminal records	Gerra G, Garofano L, Pellegrini C, Bosari S, Zaimovic A, Moi G, Avanzini P, Talarico E, Gardini F, Donnini C. Allelic association of a dopamine transporter gene polymorphism with antisocial behaviour in heroin-dependent patients. Addict Biol. 2005 Sep;10(3):275-81.
	9R allele compared to 10R	DAT1, genotype 9/9 was associated with early opiate addiction.	VNTR polymorphisms of the dopamine transporter (DAT1) gene were studied in Russian male opiate addicts. Number of addicts unknown	Galeeva AR, Gareeva AE, Iur’ev EB, Khusnutdinova EK. VNTR polymorphisms of the serotonin transporter and dopamine transporter genes in male opiate addicts. Mol Biol (Mosk). 2002 Jul-Aug;36(4):593-8.
	9R allele compared to 10R	One 9R-allele of DAT1 (compared with homozygote carriers of the 10R-allele) show heightened reactivity to drug-related reinforcement in addiction in cocaine abusers compared to controls. Because drug cues contribute to relapse, our results identify the DAT1R 9R-allele as a vulnerability allele for relapse especially during early abstinence (e.g, detoxification).	9R allele compared to 10R in N=73 human cocaine-addicted individuals and N=47 healthy controls,	Moeller SJ, ParvazMA, Shumay E, Beebe-Wang N, Konova AB, Alia-Klein N, Volkow ND, Goldstein RZ. Gene x abstinence effects on drug cue reactivity in addiction: multimodal evidence. *J Neurosci.* 2013 Jun 12;33(24):10027-36. doi: 10.1523/JNEUROSCI.0695-13.2013. PubMed PMID: 23761898; PubMed Central PMCID: PMC3682385.
	Repeat polymorphisms, including a 30-bp variable-number tandem repeat (VNTR) in intron 8 (Int8 VNTR)	Positive association was observed with allele 3 of the Int8 VNTR and cocaine abuse (allele odds ratio = 1.2, 95% confidence interval = 1.01-1.37, P = 0.036; 3/3 homozygote odds ratio = 1.45, 95% confidence interval = 1.18-1.78, P = 0.0008) The study demonstrates a robust association between cocaine dependence and a VNTR allele in SLC6A3,	Genotyped in cocaine-dependent abusers (N = 699) and in controls with no past history of drug abuse (N = 866) from Sao Paulo, Brazil.	Guindalini C, Howard M, Haddley K, Laranjeira R, Collier D, Ammar N, Craig I, O’Gara C, Bubb VJ, Greenwood T, Kelsoe J, Asherson P, Murray RM, Castelo A, Quinn JP, Vallada H, Breen G. A dopamine transporter gene functional variant associated with cocaine abuse in a Brazilian sample. *Proc Natl Acad Sci USA.* 2006 Mar 21 ;103(12):4552-7. doi: 10.1073/pnas.0504789103. Epub 2006 Mar 14. PubMed PMID: 16537431; PubMed Central PMCID: PMC1450209.
	VNTR polymorphism in the 3’ untranslated region of DAT1	Found a significantly increased prevalence of the nine-repeat allele in alcoholics compared to controls and suggested that the 9R is a major genetic determinant of vulnerability to severe alcohol withdrawal symptoms.	Analyzed a VNTR polymorphism in the 3’ untranslated region of DAT1. In N= 93 alcoholics displaying withdrawal seizures or delirium, compared with N=93 ethnically matched nonalcoholic controls	Sander T, Harms H, Podschus J, Finckh U, Nickel B, Rolfs A, Rommelspacher H, Schmidt LG. Allelic association of a dopamine transporter gene polymorphism in alcohol dependence with withdrawal seizures or delirium. Biol Psychiatry. 1997 Feb 1;41(3):299-304.
	9R allele compared to 10R	Compared to the 10R the 9 allele was associated with a greater number of drinking days	Alcohol consumption and subjective responses to alcohol in 127 young, healthy, social drinkers.	Weerts EMI, Wand GS, Mlaher B, Xu X, Stephens MIA, Yang X, MIcCaul MCE. Independent and Interactive Effects of OPRMI1 and DAT1 Polymorphisms on Alcohol Consumption and Subjective Responses in Social Drinkers. *Alcohol Clin Exp Res.* 2017 Jun;41(6):1093-1104. doi: 10.1111/acer.13384. Epub 2017 Apr 26. PubMed PMID: 28376280; PubMed Central PMCID: PMC5483245.
	40-bp variable number of tandem repeats (VNTR) ranging from 3 to 16 copies in the 3’-untranslated region (UTR) of the gene	Alcoholism phenotype was defined based on the DSM-IV criteria. The DAT1 VNTR was significantly associated with alcoholism in Badaga population but not in Kota population. Our results suggest that the A9 allele of the DAT gene is involved in vulnerability to alcoholism, but that these associations are population specific.	Genotyped the VNTR of DAT1 gene in a sample of N=206 subjects from the Kota population (111 alcohol dependence cases and 95 controls) and N=142 subjects from Badaga population (81 alcohol dependence cases and 61 controls).	Bhaskar LV, Thangaraj K, Wasnik S, Singh L, Raghavendra Rao V Dopamine transporter (DAT1) VNTR polymorphism and alcoholism in two culturally different populations of south India. Am J Addict. 2012 Jul-Aug;21(4):343-7. doi: 10.1111/j.1521-0391.2012.00244.x.
	9R of DAT1; SLC6A3) to 10R.	The frequency of individuals carrying the allele A9 [f(A9+)] was significantly higher (P = 0.01) in the group of alcoholics [f(A9+) = 0.48] compared with healthy controls [f(A9+) = 0.32].Authors suggest the allele A9 is strongly associated with alcoholism	A group of N=102 healthy subjects and = 216 alcoholics	Kohnke MD, Batra A, Kolb W, Kohnke AMI, Lutz U, Schick S, Gaertner I Association of the **dopamine transporter gene with alcoholism. Alcohol Alcohol**. 2005 Sep-Oct;40(5):339-42.
	Association between the 9-repeat allele (A9) of a 40-bp variable number tandem repeat (VNTR) polymorphism in the 3’ untranslated region (3^1^ UTR) of the SLC6A3 gene and alcoholism.	Significant associations were observed between SLC6A3 VNTR A9 and alcoholics with AWS or DT at the genotypic as well as allelic level when all ethnic populations or only Caucasian populations were considered (p< 0.05, OR 1.5-2.1).	Meta-analyses were performed for population-based case-control association studies of the SLC6A3 VNTR polymorphism with Mexican-Americans alcoholism including N=337 controls and N= 365 alcoholics	Du Y, Nie Y, Li Y, Wan YJ. The association between the SLC6A3 VNTR 9-repeat allele and alcoholism-a meta-analysis. *Alcohol Clin Exp Res.* 2011 Sep;35(9):1625-34. doi: 10.1111/j.1530-0277.2011.01509.x. Epub 2011 May 9. PubMed PMID: 21554332; PubMed Central PMCID: PMC4084904.
	A meta –analysis association of 3’-untranslated region variable-number tandem repeat (VNTR) polymorphism in SLC6A3 with alcohol dependence (AD)	Significant association of VNTR A9 genotypes with AD in all ethnic populations (pooled odds ratio [OR] 1.12; 95% confidence interval [CI] 1.00, 1.25; p = 0.045) and the Caucasian population (pooled OR 1.15; 95% CI 1.01, 1.31; p = 0.036). The authors also found VNTR A9 genotypes to be significantly associated with alcoholism as defined by the DSM-IV criteria (pooled OR 1.18; 95% CI 1.03, 1.36; p = 0.02). The authors concluded that the VNTR polymorphism has an important role in the etiology of AD, and individuals with at least 1 A9 allele are more likely to be dependent on alcohol than persons carrying the non-A9 allele	Integrating 17 reported studies with 5,929 participants including N=3,280 alcoholics and N=2,649 controls	Ma Y, Fan R, Li MD. Meta-Analysis Reveals Significant Association of the 3’-UTR VNTR in SLC6A3 with Alcohol Dependence. *Alcohol Clin Exp Res.* 2016 Jul;40(7):1443-53. doi: 10.1111/acer.13104. Epub 2016 May 24. Erratum in: Alcohol Clin Exp Res. 2017 Sep;41(9):1656-1659. PubMed PMID: 27219321; PubMed Central PMCID: PMC4930400.
**COMT**	Rs4680 Catechol-O-methyl-transferase (COMT) Val158Met	Substance-use disorder, defined by DSM-IV criteria, was associated with the Val allele for Asian samples	A total of 363 datasets were included, consisting of N=56,998 cases and N=74,668 healthy controls from case control studies, and 2,547 trios from family based studies. A meta –analysis involving 15 psychiatric disorders.	Taylor S. Association between COV1T Val158Met and psychiatric disorders: A comprehensive meta-analysis. Am J Med Genet B Neuropsychiatr Genet. 2018 Mar;177(2):199-210. doi: 10.1002/ajmg.b.32556
	*COMT* rs4680 (Val^158^Met),	*COMT* rs4680 (Val^158^Met), patients with the Val/Val genotype showed the greatest pain variability and also experienced the greatest increase in pain as a result of physical activity. These results implicate that carriers of the COMT Val will consume more opioids to reduce their exacerbated pain.	A total of 120 knee OA patients reported on their pain 3 times per day over 22 days using handheld computers, and wore an accelerometer to capture daily physical activity	Martire LM, Wilson SJ, Small BJ, Conley YP, Janicki PK, Sliwinski MJ. *COMT* and *OPRM1* Genotype Associations with Daily Knee Pain Variability and Activity Induced Pain. *Scand J Pain.* 2016 Jan 1;10:6-12. PubMed PMID: 26322144; PubMed Central PMCID: PMC4548933.
	Rs4680 Catechol-O-methyl-transferase (COMT) Val158Met	Linear regression of COMT explained the highest proportion of variance of morphine consumption (10.7%; P = .001).	Analyzed Rs4680 SNP in 201 unrelated Caucasian patients who underwent abdominal surgery and who were receiving postoperative patient-controlled analgesia-administered morphine.	De Gregori M, Diatchenko L, Ingelmo PM, Napolioni V, Klepstad P, Belfer I, Molinaro V, Garbin G, Ranzani GN, Alberio G, Normanno M, Lovisari Somaini M, Govoni S, Mura E, Bugada D, Niebel T, Zorzetto M, De Gregori S, Molinaro M, Fanelli Allegri M. Human Genetic Variability Contributes to Postoperative Morphine Consumption. J Pain. 2016 May;17(5):628-36. doi: 10.1016/j.jpain.2016.02.003.
	Rs4680 Catechol-O-methyl-transferase (COMT) Val158Met	The COMT rs4680 gene variants were different between relapse and abstinent groups. The study demonstrated that the Val increases heroin relapse.	Evaluated COMT gene polymorphism (rs4680) on relapse to heroin use during 5-year follow up. 564 heroin dependent patients were enrolled in compulsory drug rehabilitation center	Su H, Li Z, Du J, Jiang H, Chen Z, Sun H, Zhao M. Predictors of heroin relapse: Personality traits, impulsivity, COV1T gene Val158met polymorphism in a 5-year prospective study in Shanghai, China. Am J Med Genet B Neuropsychiatr Genet. 2015 Dec;168(8):712-9. doi: 10.1002/ajmg.b.32376
	Rs4680 Catechol-O-methyl-transferase (COMT) Val158Met	The rs4680 SNP showed a weak association with alcohol dependence at the allele level that did not reach significance at the genotype level. While there was significance at the allelic level a better control could have resulted in a stronger association.	The control group consisted of N=250 unrelated Caucasians (102 female and 148 male) with a mean age of 36.8 years (s.d. ±12.8 years). The control group consisted of volunteers from the general public, hospital nursing and medical staff, and university staff and students. Formal screening for psychological disorders was not undertaken in the control population. As such the controls represent an unselected control group and may include individuals with substance dependence. This represents a poorly screened control N= 120 unrelated Caucasian participants (50 female and 70 male) diagnosed as opiate-dependent were recruited for this study. All subjects were assessed using a checklist of specific criteria by a consultant psychiatrist or physician experienced in drug and alcohol dependence and met DSM-IV criteria for opiate dependence.	Voisey J, Swagell CD, Hughes IP, Lawford BR, Young RM, Morris CP. A novel SNP in COV1T is associated with alcohol dependence but not opiate or nicotine dependence: a case control study. *Behav Brain Funct.* 2011 Dec 31;7:51. doi: 10.1186/1744-9081-7-51. PubMed PMID: 22208661; PubMed Central PMCID: PMC3268714.
	Catechol-O-methyltransferase (COMT) functional polymorphism, Val158Met	In women, the COMT Val158 allele frequency was maximal in alcoholic smokers (0.85), decreasing to 0.74 in nonalcoholic smokers, 0.67 in alcoholic nonsmokers, and 0.64 in nonalcoholic nonsmokers (chi2 = 11.1, 3 df, p = 0.011). Women showed a main effect of Val 158 on smoking (p=0.003). Both male and female alcoholics were more likely to have at least 1 Vail58 allele compared with nonalcoholics (0.95 vs 0.88, p < 0.05). Approximately 30% of all participants were long-term, non-addicted light, social smokers (3.6+/−1.7 cigarettes/d); they had the same Vail 58Viet frequencies as non-smokers.	The aims of our study were firstly to investigate patterns of alcohol and tobacco consumption and comorbidity between alcoholism and smoking in Plains American Indians. Diagnostic and Statistical Manual-III-R lifetime diagnoses were assigned to 342 community-ascertained Plains American Indians (201 women, 141 men).	Enoch MA, Waheed JF, Harris CR, Albaugh B, Goldman D. Sex differences in the influence of COMT Val158Met on alcoholism and smoking in plains American Indians. Alcohol Clin Exp Res. 2006 Mar;30(3):399-406.
	A functional polymorphism (COMTVal158Met)	There was a difference in genotype frequencies between cannabis users and controls, including the distribution of the COMT genotypes (Val/Val, Val/Met) (P < 0.001) and alleles (Val vs , Met) (P< 0.01),	N=55 cannabis users and N= 75 normal controls were enrolled in this study	Baransel Isir AB, Oguzkan S, Nacak M, Gorucu S, Dulger HE, Ars1an A. The catechol-O-methyl transferase Val158Met polymorphism and susceptibility to cannabis dependence. Am J Forensic Med Pathol. 2008 Dec;29(4):320-2. doi: 10.1097/PAF.0b013e3181847e56.
	COMT Val158Met	Substance users and normal controls significantly differed in allele frequencies of COMT Val158Met (p = 0.039)	187 substance users and 386 normal controls were recruited from Northern Taiwan.	Chen CK, Lin SK, Chiang SC, Su LW, Wang LJ. Polymorphisms of COMT Val158Met and DAT1 3’-UTR VNTR in illicit drug use and drug-related psychiatric disorders. Subst Use Misuse. 2014 Sep;49(11):1385-91. doi: 10.3109/10826084.2014.901391.
	COMT 158 Val/Met	The authors found a significant excess of the high activity Val158 allele in the methamphetamine abuser group, consistent with several previous reports of association of this allele with drug abuse	A case/control design with N=416 methamphetamine abusing subjects and N= 435 normal controls.	Li T, Chen CK, Hu X, Ball D, Lin SK, Chen W, Sham PC, Loh el-W, Murray RM, Collier DA. Association analysis of the DRD4 and COMT genes in methamphetamine abuse. Am J Med Genet B Neuropsychiatr Genet. 2004 Aug 15;129B(1):120-4.
	COMT 158 Val/Met	COMT Val158Met may affect the intermediate phenotype of central dopamine receptor sensitivity. COMTVall 58Viet may confer their risk of alcohol dependence through reduced dopamine receptor sensitivity in the prefrontal cortex and hindbrain	Patients (n = 110) were alcohol dependent whereas controls (n = 99) were recruited through advertisements in regional newspapers.	Schellekens AF, Franke B, Ellenbroek B, Cools A, de Jong CA, Buitelaar JK, Verkes RJ. Reduced dopamine receptor sensitivity as an intermediate phenotype in alcohol dependence and the role of the COMT Val158Met and DRD2 Taql A genotypes. Arch Gen Psychiatry. 2012 Apr;69(4):339-48. doi: 10.1001/archgenpsychiatry.2011.1335.
	Val158Met polymorphism of the COMT gene	Found a relationship between the Val158Met polymorphism of the COMT gene and alcoholism in male subjects. Found the significant difference between male alcoholics and male controls in allele and genotype frequencies (p<0,007; and p<0,04 respectively). Confirmed the relationship between the COMT polymorphism and alcoholism in the Czech male population.	Case control study we analyzed DNA samples from N=799 subjects in total (279 male alcoholics and 120 female alcoholics, 151 male controls and 249 female controls).	Serý O, Didden W, Mikes V, Pitelová R, Znojil V, Zvolský P. The association between high-activity COMT allele and alcoholism. Neuro Endocrinol Lett. 2006 Feb-Apr;27(1-2):231-5.
**OPRM1**	A118G(rs1799971) polymorphism	Meta-analysis showed significant association between this polymorphism and susceptibility to opioid dependence in overall studies	Thirteen studies (n = 9385), comprising 4601 opioid dependents and 4784 controls	Haerian BS, Haerian MS. OPRM1 rs 1799971 polymorphism and opioid dependence: evidence from a meta-analysis. Pharmacogenomics. 2013 May;14(7):813-24. doi: 10.2217/pgs.13.57.
	OPRMI (rs1799971)	Individuals homozygous for AA at the OPRMI (rs 1799971) polymorphisms required less postsurgical opioid compared with those homozygous for GG (Hedges g, −0.270; 95% confidence interval, −0.433 to −0.108; P=0.001. Implicating more opioids required with G risk allele.	Fifty-one studies included in meta- analysis	Choi SW, Lam DMH, Wong SSC, Shiu HHC, Wang AXM, Cheung CW. Effects of Single Nucleotide Polymorphisms on Surgical and Postsurgical Opioid Requirements: A Systematic Review and Meta-Analysis. Clin J Pain. 2017 Dec;33(12):1117-1130. doi: 10.1097/AJP.0000000000000498.
	OPRMI (rs 1799971)	Ethnicity-specific meta-analyses revealed that the A118G polymorphism was significantly associated with alcohol dependence risk in Asians (GA vs. AA: odds ratio [OR], 1.73; 95% confidence interval [CI], 1.33-2.25; GA+GG vs. AA: OR, 1.57; 95% CI, 1.22-2.02), but not in Caucasians (GAvs. AA: OR, 1.05; 95% CI, 0.75-1.49; GA+GG vs. AA: OR, 1.11; 95% CI, 0.79-1.55). The OPRMI A118G polymorphism may contribute to the susceptibility of alcohol dependence in Asians	Twelve independent studies with N= 1900 cases and N=2382 controls were included. Five studies were conducted in Asians and seven in Caucasians	Chen D, Liu L, Xiao Y, Peng Y, Yang C, Wang Z. Ethnic-specific meta-analyses of association between the OPRMI A118G polymorphism and alcohol dependence among Asians and Caucasians. Drug Alcohol Depend. 2012 Jun 1;123(1-3):1-6. doi: 10.1016/j.drugalcdep.2011.10.012.
	Human μ-opioid receptor variant 118 A>G (rs1799971)	Meta-analysis of 14 studies: Cohen’s d = 0.096; p = 0.008), consisting of higher opioid dosing requirements in peri- and post-operative settings. Cohen is very conservative and as such the rs1799971 due to its small effect size, the SNP should be regarded as a part of complex genotypes underlying pain and analgesia.	Meta-analysis of 14 studies The population total was 3446 (N=1476 G carriers and 1970-on G carriers)	Walter C, Doehring A, Oertel BG, Lötsch J. μ-opioid receptor gene variant OPRMI 118 A>G: a summary of its molecular and clinical consequences for pain. Pharmacogenomics. 2013 Nov;14(15):1915-25. doi: 10.2217/pgs.13.187.
	*OPRMI mRNA*-expression in prefrontal cortex	The functional SNP allele rs1799971-A was associated with heroin addiction only in the presence of other haplotypes.	Tested 103 *OPRMI* SNPs for association with *OPRMI* mRNA expression in prefrontal cortex from 224 European Americans and African Americans of the BrainCloud cohort. We then tested the 16 putative *cis*-quantitative trait loci (*cis*-eQTL) SNPs for association with heroin addiction in the Urban Health Study and two replication cohorts, totaling **16,729** European Americans, African Americans, and Australians of European ancestry. Final analysis dataset included N =852 African Americans (307 DSM-IV-defined cases of heroin/other opioid abuse or dependence and 545 controls with no illicit drug abuse or dependence.	Hancock DB, Levy JL, Gaddis NC, Glasheen C, Saccone NL, Page GP, Hulse GK, Wildenauer D, Kelty EA, Schwab SG, Degenhardt L, Martin NG, Montgomery GW, Attia J, Holliday EG, McEvoy M, Scott RJ, Bierut LJ, Nelson EC, Krai AH, Johnson EO. Cis-Expression Quantitative Trait Loci Mapping Reveals Replicable Associations with Heroin Addiction in OPRMI. *Biol Psychiatry.* 2015 Oct 1;78(7):474-84. doi: 10.1016/j.biopsych.2015.01.003. Epub 2015 Jan 29. PubMed PMID: 25744370; PubMed Central PMCID: PMC4519434.
	OPRMI gene, All8G (rs1799971, Asn40Asp)	OPRMI A118G SNP Gil8 allele carriers showed significantly higher levels of AD severity as indicated by the MAST	N=121 Alcohol dependent (AD) patients and N= 117 healthy male subjects were included in the	Gurel ŞC, Ayhan Y, Karaaslan Ç, Akel H, Karaca RÖ, BabaoĞlu MÖ, Yaşr Ü, Bozkurt A, Dilbaz N, UluĞ BD, Demir B. μ-Opioid Receptor Gene (OPRMI) Polymorphisms A118G and C17T in Alcohol Dependence: A Turkish Sample. Turk Psikiyatri Derg. 2016 Summer;27(2):0.
	OPRMI (rs1799971) polymorphism	Research shows that there are differences in the genotypes and alleles of the OPRMI polymorphism in the case-control study	An association study of a group of Alcohol Dependent Syndrome (ADS) patients (n = 177) and control group consisted of healthy volunteers, with matched gender and age, and with psychiatric disorders excluded (η = 162).	Samochowiec A, Samochowiec J, Pelka-Wysiecka J, Kucharska-Mazur J, Grochans E, Jabłoński M, Bieńkowski P, Murawiec S, Małecka I, Mak M, Kołodziej Ł, Heitzman J, Grzywacz A. The role of OPRMI polymorphism in the etiology of alcoholism. Adv Clin Exp Med. 2019 Feb;28(2):199-202. doi: 10.17219/acem/78592.
	A single gene, OPRM1, encodes the MIOR	Results indicate that 118G allele carriers reported significantly more heroin use-related consequences and heroin-quit attempts, and were more likely to have sought treatment for their heroin use than 118AA homozygotes.	Caucasian chronic heroin users (n = 86).	Woodcock EA, Lundahl LH, Burmeister MI, Greenwald MK. Functional mu opioid receptor polymorphism (OPRMIl A(118) G) associated with heroin use outcomes in Caucasian males: A pilot study. *Am J Addict.* 2015 Jun;24(4):329-35. doi: 10.1111/ajad.12187. Epub 2015 Apr 24. PubMed PMID: 25911999; PubMed Central PMCID: PMC5541380.
	A118G rs1799971 polymorphism	When the authors assessed the associations of this polymorphism with the presence or absence of alcohol use disorder through the AUDIT score, we found that, after covariate adjustment, male carriers of the G allele had an increased risk of presenting an alcohol pattern consumption compatible with AUD (OR=2.52; 95% CI: 1.02-6.24;; *p* = 0.046). The conclusion is that these results suggest that *OPRM1* gene polymorphisms are associated with tobacco and alcohol consumption in a Spanish population, and this association could be modulated by genetic and environmental factors.	N=763 unrelated individuals (465 women, 298 men) aged 18-85 years were recruited between October 2011 and April 2012. Participants were requested to answer a 3 5-item questionnaire on tobacco and alcohol consumption, as well as to complete the AUDIT and Fagerstrom tests.	Frances F, Portoles O, Castello A, Costa JA, Verdú F. Association between Opioid Receptor mu 1 (OPRM1) Gene Polymorphisms and Tobacco and Alcohol Consumption in a Spanish Population. *Bosn J Basic Med Sci.* 15(2):31-6. doi: 10.17305/bjbms.2015.243. PubMed PMID: 26042510; PubMed Central PMCID: PMC4469933.
GABRB3	GABRB3 promotor Alpha-3 CA-repeat 181 (downstream) CHr15	Data suggest that GABRB3 might be associated with heroin dependence, and increased expression of GABRB3 might contribute to the pathogenesis of heroin dependence.	A case-control association analysis between N=576 subjects with heroin dependence (549 males, 27 females) and 886 controls (472 males, 414 females)	Chen CH, Huang CC, Liao DL. Association analysis of GABRB3 promoter variants with heroin dependence. *PLoS One.* 2014 Jul 15;9(7):e102227. doi: 10.1371/journal.pone.0102227. PubMed PMID: 25025424; PubMed Central PMCID: PMC4098998.
	Chr-15-RS2677918 9(Upstream) Risk CT	For this allele it was found that the BRBR3 significantly associated with both heroin and cocaine risk (P<.003). This study suggests numerous potential susceptibility loci (or markers that tag them) in the glutamatergic and GABAergic pathways for heroin and cocaine addiction, in subjects of African and European ancestry, with partial overlap in susceptibility loci between populations and between addictions to different drugs.	Total population N=1860; N=1142=Heroin subjects; and 439 controls ; Eastern European / middle Eastern = N= 827 Heroin and 232 Controls; African-American (AA) =315 heroin and 207 controls; AA=279 Cocaine Total 1860 = 1142 h; 279 cocaine and 439 controls	Levran O, Peles E, Randesi MI, Correa da Rosa J, Ott J, Rotrosen J, Adelson MI, Kreek MIJ. Glutamatergic and GABAergic susceptibility loci for heroin and cocaine addiction in subjects of African and European ancestry. *Prog Neuropsychopharmacol Biol Psychiatry.* 2016 Jan 4;64:118-23. doi: 10.1016/j.pnpbp.2015.08.003. Epub 2015 Aug 12. PubMed PMID: 26277529; PubMed Central PMCID: PMC45 64302.
	Receptor beta3 subunit (GABRB3) 181 variant	When the DRD2 and the GABRB3 variants are combined, the risk for alcoholism is more robust than when these variants are considered separately.	Determined in a population-based association study of Caucasian non-alcoholic and alcoholic subjects. In severe alcoholics, compared to non-alcoholics	Noble EP, Zhang X, Ritchie T, Lawford BR, Grosser SC, Young RMI, Sparkes RS. D2 dopamine receptor and GABA(A) receptor beta3 subunit genes and alcoholism. Psychiatiy Res. 1998 Nov 16;81(2):133-47.
	GABRB3 CHR 15	All combinations of genes, a count of the number of hypodopaminergic genotypes. Hypodopaminergic functioning predicted drug use in males; however, in females, a deleterious environment was the salient predictor. This preliminary study suggests that it is possible to identify children at risk for problematic drug use prior to the onset of drug dependence.	Variables included in the study were dopaminergic genes (ANXK1 TaqI A, DRD2 C957T, DRD4 7R, COMT Val/Met substitution, and SLC6A3 9R) and a GABAergic gene (GABRB3). Genotyping adolescent Caucasian children of alcoholics (N=57 males, N =54 females; mean age = 14.5 years)	Conner BT, Hellemann GS, Ritchie TL, Noble EP. Genetic, personality, and environmental predictors of drug use in adolescents. J Subst Abuse Treat. 2010 Mar;38(2):178-90. doi: 10.1016/j.jsat.2009.07.004.
**MAOA**	30 BP VNTR-3.5R, 4R DN repeat polymorphisms	Significant associations of alcohol dependence with MAOA alleles (RFLP and DNRP) were found among the Han Chinese. It is concluded that MAOA mutations may play a role in susceptibility to alcoholism among Han Chinese	Male alcoholic patients and nonalcoholic comparison subjects among Han Chinese	Hsu YP, Loh EW, Chen WJ, Chen CC, Yu JMI, Cheng AT. Association of monoamine oxidase A alleles with alcoholism among male Chinese in Taiwan. Am J Psychiatry. 1996 Sep;153(9):1209-11.
	30 BP VNTR-3.5R, 4R DN repeat polymorphisms	High activity 4-repeat allele frequency Buss Durkee Hostility Inventory (BDHI) mean total scores were significantly higher in heroin addicts than in controls (p<0.001)	N=199 male subjects of Italian descent, a sample comprising 95 healthy subjects and N= 104 heroin-dependent subjects	Gerra G, Garofano L, Bosari S, Pellegrini C, Zaimovic A, Moi G, Bussandri M, Moi A, Brambilla F, Mameli A, Pizzamiglio M, Donnini C. Analysis of monoamine oxidase A (MAO-A) promoter polymorphism in male heroin-dependent subjects: behavioural and personality correlates. J Neural Transm (Vienna). 2004 May;111(5):611-21.
	Dinucleotide repeat length polymorphism at the MAOA	A significant correlation between the presence/absence of the disorder and the length of the MAOCA-1 repeat was found in males, but not females, with “long” alleles (repeat length above 115 bp) associated with both increased risk for the disorder and lower age of onset of substance abuse.	An association between the liability to early onset alcoholism/substance abuse and a recently discovered dinucleotide repeat length polymorphism at the MAOA gene (MAOCA-1) was examined using polymerase chain reaction (PCR).	Vanyukov MM, Moss HB, Yu LM, Tarter RE, Deka R. Preliminary evidence for an association of a dinucleotide repeat polymorphism at the MAOA gene with early onset alcoholism/substance abuse. Am J Med Genet. 1995 Apr 24;60(2):122-6.
	High activity allele (L: 4 repeats (4R)) of MAOA-LPR	MAOA-LPR may have predictive effects on co-morbid BPD in female heroin-dependent patients;	N=296 female heroin-dependent patients (including 61 patients with Borderline Personality Disorder(BPD )and 235 without BPD) and N= 101 normal females	Yang M, Mamy J, Wang Q, Liao YH, Seewoobudul V, Xiao SY, Hao W. The association of 5-HTR2A-1438A/G, COMTVal158Met, MAOA-LPR, DATVNTR and 5-HTTVNTR gene polymorphisms and borderline personality disorder in female heroin-dependent Chinese subjects. Prog Neuropsychopharmacol Biol Psychiatry. 2014 Apr 3;50:74-82. doi: 10.1016/j.pnpbp.2013.12.005.
	Transcript factor TFAP2b regulates MAOA	Results showed that the high-functioning allele was significantly more common among the female alcoholics, compared to the nonalcoholic controls. This suggest that high allele would up-regulate MAO-A Long 3.5 to 4R	Compared a sample of female alcoholics (n=107), sentenced to institutional care for their severe addiction, contrasted against a control sample of adolescent females (n=875)	Nordquist N, Gokturk C, Comasco E, Nilsson KW, Oreland L, Hallman J. Transcription factor AP2 beta involved in severe female alcoholism. Brain Res. 2009 Dec 11;1305 Suppl:S20-6. doi: 10.1016/j.brainres.2009.09.054
**SLC6A4(5HTTLPR)**	43BP-INDEL/VNTR CHR17-rs25531 LG, S	By univariate ANOVA, a statistically significant difference was found in the onset of AD: the mean age of onset resulted to be of 25.4 years in males in respect to 28.1 in females. In particular in males, the early AD onset was different, in a statistically significant manner, depending on the presence of at least one S.	Genotyping of the 5-HTTLPR (L/S) and rs25531 (A/G) polymorphisms of the SLC6A4 gene was performed on N= 403 alcoholics outpatients and N=427 blood donors.	Pascale E, Ferraguti G, Codazzo C, Passarelli F, Mancinelli R, Bonvicini C, Bruno SM, Lucarelli M, Ceccanti M. Alcohol dependence and serotonin transporter functional polymorphisms 5-HTTLPR and rs25531 in an Italian population. Alcohol Alcohol. 2015 May;50(3):259-65. doi: 10.1093/alcalc/agv014.
	promoter region (5-HTTLPR) (rs25531)	The genotype frequencies of the 5-HTTLPR S+ (S/S, S/LG, LG/LG) polymorphisms were significantly higher in opiate-dependent patients ( p = 0.01). There was a significant interaction between the TPH1 A218C A/C and 5-HTTLPR S+ gene polymorphisms in opiate-dependent (OR 2.72, p = 0.01),	Opiate-dependent patients (n = 309), and healthy controls (n = 301) were recruited from the Han Chinese population in Taiwan.	Wang TY, Lee SY, Chung YL, Chen SL, Li CL, Chang YH, Wang LJ, Chen PS, Chen SH, Chu CH, Huang SY, Tzeng NS, Hsieh TH, Lee IH, Chen KC, Yang YK, Hong JS, Lu RB. TPH1 and 5-HTTLPR Genes Specifically Interact in Opiate Dependence but Not in Alcohol Dependence. Eur Addict Res. 2016;22(4):201-9. doi: 10.1159/000444676.
	rs25531 polymorphisms of SLC6A4 gene	The biallelic analysis revealed that the frequency of 5-HTTLPR (LL/LS/SS) genotypes was statistically significant different between drug-dependent individuals and controls ( p = 0.04)	Jordanian drug male addicts of Arab descent (n = 192) meeting the Diagnostic and Statistical Manual of Mental Disorders - Fourth edition criteria for drug dependence and 230 healthy male controls from an ethnically homogenous Jordanian Arab population were examined.	Al-Eitan LN, Jaradat SA, Qin W, Wildenauer DM, Wildenauer DD, Hulse GK, Tay GK. Characterization of serotonin transporter gene (SLC6A4) polymorphisms and its association with drug dependence in a Jordanian Arab population. Toxicol Ind Health. 2014 Aug;30(7):598-610. doi: 10.1177/0748233712462446.

**Table 3.** Global Heterozygous prevalence

SNP	Global Heterozygous Prevalence
rs4532	32%
rs1800497	46%
rs6280	41%
rs1800955	Frequency of C allele=0.42 Prevalence not available
rs4680	42%
rs1799971	29%

**Table 4.** Prevalence in terms of N and different ethnic groups

Frequency of Risk Allele (n)												
	COMT	DRD1	DRD2	DRD3	DRJD4	OPRM1	DAT1-	MAOA	GABRB3	DRD4-	HTTLPR	Total Tested
							Repeats			Repeats		
**Asian**	5	9	3	5	4	5	0	3	3	1	9	9
**Black or**	18	40	23	39	24	1	8	16	8	13	30	42
**African American**												
**Hispanic or Latino**	20	25	17	15	16	8	0	20	18	19	22	29
**Mixed Race**	0	1	1	1	1	0	0	0	1	0	1	1
**Other**	6	9	2	6	7	0	0	5	4	4	5	9
**Unknown**	26	30	11	20	21	14	0	24	20	9	29	36
**White or Caucasian**	224	245	100	157	206	61	4	226	191	88	222	293

**Table 5.** Prevalence in terms of % across and different ethnic groups

Frequency of Risk Allele (%)												
	COMT	DRD1	DRD2	DRD3	DRJD4	OPRM1	DAT1-	MAOA	GABRB3	DRD4-	HTTLPR	Total Tested
							Repeats			Repeats		
**Asian**	56%	100%	33%	56%	44%	56%	0%	33%	33%	11%	100%	9
**Black or African American**	43%	95%	55%	93%	57%	2%	19%	38%	19%	31%	71%	42
**Hispanic or Latino**	69%	86%	59%	52%	55%	28%	0%	69%	62%	66%	76%	29
**Mixed Race**	0%	100%	100%	100%	100%	0%	0%	0%	100%	0%	100%	1
**Other**	67%	100%	22%	67%	78%	0%	0%	56%	44%	44%	56%	9
**Unknown**	72%	83%	31%	56%	58%	39%	0%	67%	56%	25%	81%	36
**White or Caucasian**	76%	84%	34%	54%	70%	21%	1%	77%	65%	30%	76%	293

## Abbreviations:

SNP: Single Nucleotide Polymorphism
VNTRs: Variable Number Tadem Report
